# Saturated and monounsaturated fatty acids in membranes are determined by the gene expression of their metabolizing enzymes SCD1 and ELOVL6 regulated by the intake of dietary fat

**DOI:** 10.1007/s00394-019-02121-2

**Published:** 2019-11-01

**Authors:** Kathrin Weiss-Hersh, Ada L. Garcia, Tamás Marosvölgyi, Mónika Szklenár, Tamás Decsi, Ralph Rühl

**Affiliations:** 1 Department of Biochemistry and Molecular Biology, University of Debrecen, Debrecen, Hungary; 2 Human Nutrition, School of Medicine, College of Medical, Veterinary and Life Sciences, University of Glasgow, Glasgow, UK; 3 Department of Paediatrics, University of Pécs, Pécs, Hungary; 4 Paprika Bioanalytics BT, Debrecen, Hungary

**Keywords:** Fish oil, Olive oil, Margarine, Coconut fat, Sunflower oil, Hepatic gene expression

## Abstract

**Purpose:**

We investigated the effect of dietary fats on the incorporation of saturated (SAFAs) and monounsaturated dietary fatty acids (MUFAs) into plasma phospholipids and the regulation of the expression of lipid-metabolizing enzymes in the liver.

**Methods:**

Mice were fed different diets containing commonly used dietary fats/oils (coconut fat, margarine, fish oil, sunflower oil, or olive oil) for 4 weeks (*n* = 6 per diet group). In a second experiment, mice (*n* = 6 per group) were treated for 7 days with synthetic ligands to activate specific nuclear hormone receptors (NHRs) and the hepatic gene expression of CYP26A1 was investigated. Hepatic gene expression of stearoyl-coenzyme A desaturase 1 (SCD1), elongase 6 (ELOVL6), and CYP26A1 was examined using quantitative real-time PCR (QRT-PCR). Fatty acid composition in mouse plasma phospholipids was analyzed by gas chromatography (GC).

**Results:**

We found significantly reduced hepatic gene expression of SCD1 and ELOVL6 after the fish oil diet compared with the other diets. This resulted in reduced enzyme-specific fatty acid ratios, e.g., 18:1n9/18:0 for SCD1 and 18:0/16:0 and 18:1n7/16:1n7 for ELOVL6 in plasma phospholipids. Furthermore, CYP26A1 a retinoic acid receptor-specific target was revealed as a new player mediating the suppressive effect of fish oil-supplemented diet on SCD1 and ELOVL6 hepatic gene expression.

**Conclusion:**

Plasma levels of MUFAs and SAFAs strongly reflect an altered hepatic fatty acid-metabolizing enzyme expression after supplementation with different dietary fats/oils.

## Introduction

Fatty acids of nutritional relevance can be grouped as saturated fatty acids (SAFAs), monounsaturated fatty acids (MUFAs), and polyunsaturated fatty acids (PUFAs). These fatty acids are found in commonly consumed dietary fats such as coconut fat, rich in SAFAs; olive oil, rich in MUFAs [mainly oleic acid (OLA, 18:1n9)]; fish oil, rich in n3-PUFAs [mainly eicosapentaenoic acid (EPA, 20:5n3) and docosahexaenoic acid (DHA, 22:6n3)]; sunflower oil, rich in n6-PUFAs [mainly linoleic acid (LA, 18:2n6)]; and margarine, a mix of n6-PUFAs, MUFAs, and SAFAs.

Current dietary recommendations for chronic disease prevention are to focus on the quality of dietary fat consumed favoring MUFA and PUFA sources while limiting SAFA-rich diets [1–3]. However, high intake of n6-PUFAs results in a shift of the n3/n6 PUFA ratio towards a more pronounced n6-PUFAs ratio, which has been associated with the pathogenesis of various chronic inflammatory diseases [4]. Another alternative to reduce SAFA intake is consumption of margarine with a higher PUFAs’ and MUFAs’ content; however, the benefits of margarine consumption are not conclusive [5–7]. Thus, a better understanding of the roles fatty acids play on mechanisms underpinning health and disease is needed.

Besides providing an energy source for metabolic processes, fatty acids have the potential to act as regulators of gene expression of enzymes in lipid homeostasis, fatty acid metabolism for energy homeostasis, and inflammation via nuclear hormone receptor (NHR)-mediated signaling [8]. Fatty acids, as well as specific synthetic ligands can activate NHR-mediated signaling such as retinoid X receptor (RXR)-, liver X receptor (LXR)-, peroxisome proliferator-activated receptors (PPARs)-, and retinoic acid receptor (RAR)-mediated signaling. These receptors are major regulators of genes associated with fat and energy metabolism [9–13].

A further important fate of essential fatty acids is their incorporation into phospholipids, which are key components of cell membranes [8, 14]. In humans, the phospholipid fatty acid composition is well associated with dietary fat intake [15–17]. The composition of phospholipids adapts depending of the type of dietary fatty acids consumed because of a constant turnover. This adaptation following fatty acid change could also alter the functionality and stability of affected cellular membranes [8, 18]. The availability of fatty acids to be incorporated into phospholipids also depends on the enzymatic activity of fatty acid-metabolizing enzymes [19].

The enzymes stearoyl-desaturase 1 (SCD1) and elongase 6 (ELOVL6) are key regulators of MUFA and SAFA content in phospholipids [20, 21]. SCD1 desaturates stearic acid (STE, 18:0) or palmitic acid (PAL, 16:0) to 18:1n9 or palmitoleic acid (PAM, 16:1n7), respectively. ELOVL6 further elongates 16:0–18:0 and 16:1n7 to vaccenic acid (VAC, 18:1n7) [21]. Our previous studies in mice have shown that diet modulates the hepatic gene expression of SCD1. A diet rich in sunflower oil, and its major fatty acid constituents (n6-PUFAs) was an effective suppressor of the hepatic SCD1 and ELOVL6 expression [22]. This suppression was also observed in phospholipid composition concomitantly with a significant reduction of their metabolic products measured in cellular membranes of liver tissue, in particular MUFAs [22]. The mechanism of suppression of SCD1 and ELOVL6 by dietary fats with a high n6-PUFA content is still not fully understood, while it is well known that dietary n3-PUFAs also have a pronounced suppressive effect on the expression of these enzymes [23]. Regulation of the gene expression of SCD1 and ELOVL6 requires activation of RXR, a nuclear hormone receptor activated mainly by forms of vitamin A [24–26].

A novel theory is that CYP26A1, a well-known target gene of RAR-mediated signaling [27], is also implicated as a feedback mechanism in degradation of the endogenous ligands of the RARs, all-*trans*-retinoic acid towards less active hydroxy-/oxo-retinoic acids [27, 28]. The clearance of the endogenous RXR ligand [29] is likely also due to CYP26A1, an enzyme induced by 22:6n3, one of the main n3-PUFAs in fish oil [29, 30]. As a logical consequence, the activity of RXR is dependent on the availability of its natural derived ligands, which  in turn is regulated by the enzymatic activity of CYP26A1. These mechanisms could explain the suppressive effect of fish oil on the gene expression of SCD1 and ELOVL6 [31], but this needs testing.

The aim of this study was (1) to determine the impact of nutritionally relevant dietary fats (coconut fat, margarine, olive oil, sunflower oil, and fish oil) on the hepatic gene expression of major fat metabolizing enzymes and on ratios and amounts of major fatty acids present in plasma; (2) to evaluate the hepatic expression of major fat metabolizing enzymes SCD1 and ELOVL6 and their activity represented by major substrate vs. product ratios; and (3) to investigate the hepatic gene expression of CYP26A1 after treatment with synthetic ligands of specific nuclear hormone receptors (NHRs) such as liver X receptor (LXR), peroxisome proliferator-activated receptors (PPARs), RXR, and RARs. CYP26A1 is considered as a possible candidate for indirect regulation of the hepatic gene expression of SCD1 and ELOVL6. We hypothesized that these mechanisms are modulated by retinoid-mediated signaling.

## Materials and methods

Animal experiments were performed at the Laboratory Animal Core Facility of the University of Debrecen (Debrecen, Hungary) in accordance with the ethical guidelines of Hungary. Ethical approval was obtained from the Hungarian Animal Experimental Scientific Ethics Council in Budapest (registration 25/2006/DE MÁB). The Animal Experimentation Commission of University of Debrecen also approved the experiment.

### Animal studies

Six-to-eight-week-old female standard C57BL6 (Strain Code 027) mice, purchased from Charles River (Budapest, H), were first fed ad libitum for 2 weeks with regular chow (VRF1, Altromin GmbH, Lage, D).

#### Dietary fats and oil supplementation study

After the acclimatization period of 2 weeks, the animals were divided into five groups (total *n* = 30, 6 animals per diet group) and were assigned to each receive a different type of dietary fat for 4 weeks. The sample size was based on our previous experiments that tested the effect of ligand and dietary fats on SCD1 expression [22, 24]. These studies were powered, 99% and 72%, respectively, to detect a five- and threefold increase in SCD1 expression in experimental vs. control conditions with a level of *p* < 0.05.

#### Oil and fat supplementation diets

The experimental diets were formulated according to Bonilla et al. [32] and contained 415 g/kg diet (41.5%) wheat starch (Weizenstärke FOODSTAR, provided by Kröner-Stärke Ibbenbüren, D), 280 g/kg diet (28.0%) sucrose (purchased from a local supermarket), 180 g/kg diet (18.0%) casein from bovine milk (purchased from Sigma-Aldrich, Budapest, H), and 20 g/kg diet (2%) cellulose VIVAPUR (provided by JRS Pharma GmbH & Co. KG, Rosenberg, D). The content of minerals was 45 g/kg diet (4.5%) (Mineral-Spurenelemente-Vormischung C1000). The vitamin content was 10 g/kg diet (1%) (Vitamin-Vormischung C1000) and was purchased from Altromin GmbH (Lage, D). The source of dietary fat in each experimental diet differed, while the content remained the same in each diet at 50 g fat/kg diet (5.0%) which is considered a normal fat content in laboratory animal-based feeding studies [32]. The fat sources were sunflower oil, fish oil, coconut fat, and olive oil (provided by Henry Lamotte, Bremen, D) and margarine (purchased from a local supermarket). The carbohydrate/protein/fat ratio in percent was 69.5%/18%/5%, and other nutrients made up the remaining 7.5%. Table 1 displays the fatty acid composition of each diet. The composition of fatty acids for all dietary fats and of phospholipids was analyzed by GC. All values were normalized to coconut fat (= 1) to compare diet groups with each other and to compare GC values of diet and phospholipids to each other.

**Table 1:** Composition of selected fatty acids of experimental diets analyzed by GC and displayed as weight %

	COC	MAR	OLI	SUN	FIS
**SAFA**	93.35	31.34	15.24	11.59	31.58
16:0	18.21	21.24	11.9	6.47	20.56
18:0	20.47	3.51	2.7	4.07	4.11
**MUFA**	2.43	44.58	73.56	19.98	24.77
16:1n7	0.06	0.24	1.21	0.02	8.3
18:1n7	0.1	1.92	1.79	0.47	3.22
18:1n9	2.16	40.89	70.4	19.44	10.51
20:1n9	0.08	0.85	0.17	0.05	1.82
**n6-PUFA**	3.7	18.97	10.63	68.17	5.94
18:2n6	3.23	18.69	10.52	67.86	3.51
18:3n6	< 0.001	0.01	< 0.001	< 0.001	0.26
20:3n6	< 0.001	< 0.001	< 0.001	0.04	0.19
20:4n6	< 0.001	0.02	< 0.001	0.06	1.02
22:4n6	0.02	0.02	< 0.001	< 0.001	0.11
22:5n6	< 0.001	0.03	< 0.001	< 0.001	0.46
**n3-PUFA**	0.32	5.03	0.49	0.20	37.14
18:3n3	0.29	4.79	0.45	0.17	1.29
18:4n3	< 0.001	0.14	0.02	< 0.001	3.09
20:5n3	< 0.001	0.03	0.02	0.03	16.11
22:5n3	0.02	< 0.001	< 0.001	< 0.001	2.77
22:6n3	0.02	0.02	< 0.001	0	13.72

*COC* coconut fat, *MAR* margarine, *OLI* olive oil, *SUN* sunflower oil, *FIS* fish oil

#### Nuclear hormone receptor-specific ligand study [24]

After the acclimatization period of 2 weeks, animals (total n = 42, 6 animals per treatment group, 7 treatment groups including vehicle) were gavaged daily for 1 week with nuclear hormone receptor-specific synthetic ligands dissolved in 25% Cremophor EL (Sigma-Aldrich, Budapest, H)/water (v/v). The base diet of these animals was standard chow (VRF1, Altromin, D). The vehicle (Cremophor EL) was applied at 5 ml/kg body weight (b. w.). Rosiglitazone a PPARγ ligand was bought from Biomol (Butler Pike, USA), applied dosage 3 mg/kg b.w. [33] and LG268 a RXR ligand, applied dosage 30 mg/kg b.w. [34] was a gift from Ligand Pharmaceuticals (San Diego, Calif., USA). AM580 (RAR ligand, applied dosage 10 mg/kg b.w.) [35], GW7647 (PPARα ligand, applied dosage 3 mg/kg b.w.) [36], and GW0742 (PPAR β/δ ligand, applied dosage 5 mg/kg b.w.) [37], were purchased from Biotrend Chem. GmbH (Köln, D) and T0901317 (LXR ligand, applied dosage 20 mg/kg b.w.) [38] from Cayman Chemical Company (Tallinn, EST).

#### Animal handling

All mice had free access to water and food for the duration of the experiment. They were kept at 22 °C room temperature with a 12 h day/night cycle. All animals were killed by anaesthesia with halothane. Blood collection was carried out by cardiac puncture. The blood was centrifuged for 10 min and plasma was stored at − 80 °C. The mice were dissected, and liver samples were weighed and immediately frozen in liquid nitrogen and later stored at − 80 °C.

### RNA isolation from liver and QRT-PCR

Total RNA was isolated from liver and quantified by QRT-PCR (quantitative real-time PCR). Samples of liver tissue (50 mg) were homogenized in Trizol (10 mg tissue/100 µl Trizol, Sigma-Aldrich, Budapest, H) and extracted with chloroform (20 µl/100 µl Trizol). The aqueous phase was mixed with 700 µl of ethanol (70% v/v) and loaded on the RNA isolation column (GenElute Mammalian Total RNA Miniprep Kit, Sigma-Aldrich, Budapest, Hungary). RNA was isolated from tissue according to the protocol of Sigma-Aldrich and eluted in nuclease free water. Concentration and purity of RNA were measured by Nanodrop (Thermo, Budapest, Hungary). cDNA was obtained by reverse transcription (10 min 25 °C, 120 min 42 °C, 5 min 72 °C) and amplified via QRT-PCR (40 cycles: 12 s 94 °C, 45 s 60 °C, 60 s 94 °C). The primer and probe for the expression analysis (Taq-Man-Gene Expression Assay) as well as the quantitative real-time PCR detection system (ABI-PRISM, 7900HT Sequence Detection System) were purchased from Applied Biosystems (Budapest, Hungary). The expression of genes was normalized to cyclophilin A (house-keeping gene): primer 77“+”5´-CGATGACGAGCCCTTGG-3´, primer 142”-“5´-TCTGCTGTCTTTGGAACTTTGTC-3´, probe (69+ , 96 +): FAM-CGCGTCTCCTTCGAGCTGTTTGCA, quencher tetramethylrhodamine (TAMRA). The amplification signal was detected and analyzed by the SDS2.1 program from Applied Biosystems, Budapest, Hungary. The expression of the house-keeping gene was stable and was not influenced by dietary treatment. The hepatic gene expression of target genes SCD1, ELOVL6, and CYP26A1 was normalized to coconut fat supplementation diet (= 1).

### Fatty acid analysis by gas chromatography (GC)

Food and plasma samples were analyzed by GC. Frozen samples were thawed and the pentadecanoylphosphatidylcholine (L-α phosphatidylcholine, dipentadecanoyl, Sigma-Aldrich, Budapest, Hungary) internal standard was added. Lipids were extracted by the addition of 3 ml chloroform and 1 ml methanol according to the method of Folch [39]. The mixture was vortexed at 3000 rpm for 15 min. The lower layer was then aspirated into vials and evaporated under an N_2_ stream. Lipid extracts were reconstituted in 70 µl chloroform and lipid classes were separated by thin-layer chromatography (TLC). The solvent mix for TLC of plasma lipids was as follows: hexane: diethyl ether: chloroform: acetic acid (21:6:3:1, v/v). The bands were stained with dichlorofluorescein and removed by scraping and transesterified in 1 ml of 3 N HCl in methanol solution (3 N methanolic HCl, Supelco, Budapest, H) at 84 °C for 45 min [40]. For plasma samples, the phospholipid fraction was analyzed after TLC separation. Fatty acids were analyzed by high-resolution capillary GC using a Finnigan 9001 gas chromatograph (Finnigan/Tremetrics Inc., Austin, TX, USA) with split injection (ratio 1:25), an automatic sampler (A200SE; CTC Analytic, Zwingen, CH, USA), and a flame ionization detector with a DB-23 cyanopropyl column of 40 m length (J&W Scientific, Folsom, CA, USA). The temperature program was set to the following parameters: temperature of the injector at 80 °C/min up to 280 °C, temperature of the detector at 280 °C, temperature of the column area at 60 °C for 0.2 min, temperature increase by 40 °C/min up to 180 °C, 5 min isothermal period, temperature increase by 1.5 °C/min up to 200 °C, 8.5 min isothermal period, temperature increase by 40 °C/min up to 240 °C, and 13 min isothermal period. The constant linear velocity was 0.3 m/s (referred to 100 °C). Peak identification was confirmed by comparison with authentic mixtures of weighed fatty acid methylesters (GLC-463: Nu-Chek Prep, Elysian, MN, USA; and Supelco 37 FAME Mix: Supelco, Bellefonte, PA, USA). Individual fatty acid responses determined from weighed standards were used to calculate the percentage by weight for each fatty acid (between 12 and 24 carbon atoms) based on the area under the curve. Values were normalized to coconut fat (= 1) and compared with the other diet groups. The normalized values of enzyme-specific fatty acids for SCD1 and ELOVL6 analyzed by GC are displayed for diet and phospholipids. Normalization of values of the enzyme-specific fatty acid profile in diet and phospholipids enabled a direct comparison.

### Graphical analysis

Network graphical analysis was created using the mixOmics package in R (3.5 version) [41]. The resulting color codes for the mouse data as well as the food data were incorporated into a self-constructed graphic representation.

### Statistical analysis

Results are shown as mean and standard error of mean. The effect of diet (comparing all diet groups to each other) was analyzed using the Kruskal–Wallis test followed by paired analysis. All statistical analyses were done using SPSS (15.0) software (SPSS Inc., Chicago, USA). Statistically significance was accepted at *p* < 0.05.

## Results

### MUFA/SAFA content in phospholipids

#### Role of SCD1 on fatty acid composition

##### Concentrations of SCD1-dependent fatty acids and specific fatty acid ratios measured in diet

**Fig. 1:**
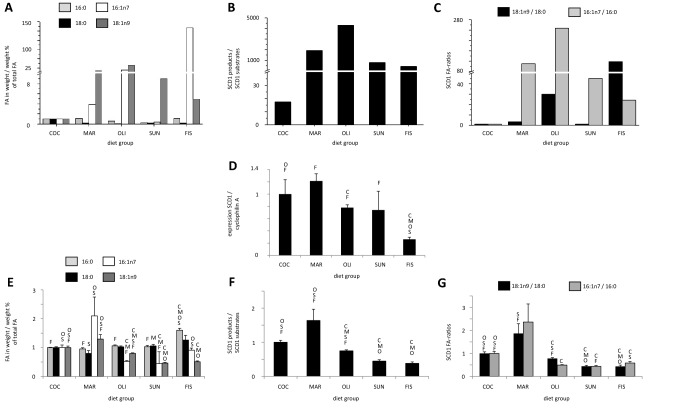
**a** SCD1 dependent fatty acids in diets, in weight/weight %, **b** fatty acid ratios (products 16:1n7, 18:1n9 vs. substrates 16:0, 18:0) of SCD1) in experimental diets, **c** specific SCD1 ratios (16:1n7/16:0, 18:1n9/18:0) of fatty acids in experimental diet groups, **d**) hepatic gene expression of SCD1, **e** SCD1 dependent fatty acids in phospholipids, in weight/weight %, **f** fatty acid ratios (products 16:1n7, 18:1n9 vs. substrates 16:0, 18:0) in phospholipids of experimental diet groups, **g** specific SCD1 ratios (16:1n7/16:0, 18:1n9/18:0) of fatty acids in phospholipids. Fatty acid composition of diet and phospholipids in plasma was analyzed by GC. Hepatic gene expression of SCD1 was analyzed by QRT-PCR. All values were normalized to coconut diet representing 1 and statistical difference was accepted for *p* < 0.05, *COC (C)* coconut fat, *MAR (M)* margarine, *OLI (O)* olive oil, *SUN (S)* sunflower oil, *FIS (F)* fish oil

The fatty acids of the SCD1 metabolic pathway (16:0, 16:1n7, 18:0, 18:1n9) in all diet groups were analyzed. Fatty acid comparisons between all diet groups showed that the highest amount of 16:0 was found in the margarine diet, while the lowest amount was measured in the sunflower oil diet (Fig. 1a). The highest concentrations of 16:1n7 were measured in the fish oil diet and the lowest in the sunflower oil diet (Fig. 1a). For 18:0, the highest concentration was found in the coconut fat diet and the lowest in the olive oil diet (Fig. 1a). 18:1n9 was highest in the olive oil diet and lowest in the coconut fat diet (Fig. 1a). The ratio of substrates and products of SCD1 was highest in the olive oil diet and lowest in the coconut fat diet (Fig. 1b). The ratio of 16:1n7/16:0 was highest in the fish oil diet and lowest in the coconut fat diet. The ratio of 18:1n9/18:0 was highest in the olive oil diet and lowest in the coconut fat diet (Fig. 1c).

##### Effects of diets on the hepatic SCD1 expression

The hepatic gene expression of SCD1 was highest after the margarine diet and lowest after the fish oil diet (Fig. 1d). Significant differences could be observed in the hepatic gene expression between the different diets with statistically significant effects between the fish oil and coconut fat, margarine, and olive oil diets (*p* < 0.05). Furthermore, the hepatic gene expression of SCD1 was significantly higher after the margarine fat diet compared with the olive oil and fish oil diet (*p* < 0.05).

##### Relative concentrations of fatty acids and fatty acid ratios in phospholipids

In plasma phospholipids, 16:0 and 18:0 were found in highest concentrations in animals fed the fish oil diet, while the lowest concentrations were found in animals which consumed the margarine diet (Fig. 1e). Levels of 18:1n9 and 16:1n7 were highest in animals fed the margarine diet and lowest after the sunflower oil and fish oil diet (Fig. 1e). The combined substrate/product ratio of SCD1 was highest after the margarine diet; this was significantly higher than for olive oil, sunflower oil and fish oil diets (*p* < 0.05) (Fig. 1f). The lowest substrate/product ratio of SCD1 in phospholipids was for the fish oil diet group which was significantly different to the coconut fat, margarine, and olive oil diets (*p* < 0.05). The specific ratios of 16:1n7/16:0 and 18:1n9/18:0 were highest after the margarine diet (Fig. 1g); the 18:1n9/18:0 ratio was significantly higher in the margarine diet compared with the sunflower and fish oil diets. The lowest ratio of 16:1n7/16:0 was found in the sunflower diet which was significantly lower compared with the coconut fat and fish oil diets (*p* < 0.05). The lowest value of 18:1n9/18:0 was observed in the fish oil diet; this was significantly lower than the ratios observed in the coconut fat, margarine, and olive oil diets (Fig. 1g).

In summary, the relative concentrations of SAFAs 16:0 and 18:0 as well as MUFAs 16:1n7 and 18:1n9 measured in the experimental diets were different from those measured in plasma phospholipids. Furthermore, the SCD1-specific fatty acid ratio of 18:1n9/18:0 detected in phospholipids, and to a lesser extent, the 16:1n7/16:0 ratio reflected the hepatic gene expression of this enzyme.

#### Role of ELOVL6 on fatty acid composition

##### Concentrations of ELOVL6-dependent fatty acids and specific fatty acid ratios measured in diet

**Fig. 2:**
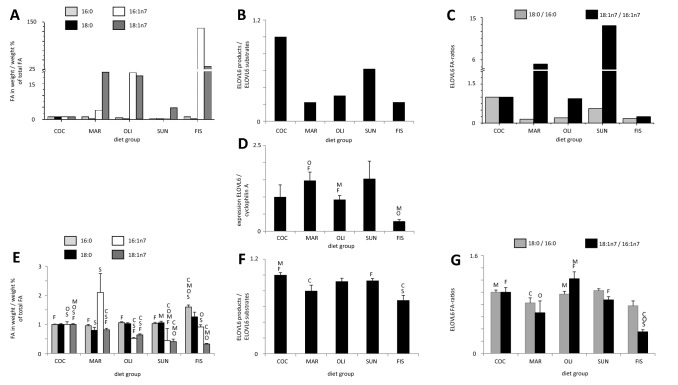
**a** ELOVL6 dependent fatty acids in diets, in weight/weight %, **b** fatty acid ratios (products 18:1n7, 18:0 vs. substrates 16:1n7, 16:0 of ELOVL6) in experimental diets, **c** specific ELOVL6 ratios (18:0/16:0, 18:1n7/16:1n7) of fatty acids in experimental diet groups, **d** hepatic gene expression of ELOVL6, **e** ELOVL6-dependent fatty acids in phospholipids, in weight/weight %, **f** fatty acid ratios (products 18:1n7, 18:0 vs. substrates 16:1n7, 16:0 of ELOVL6) in phospholipids of experimental diet groups, and **g** specific ELOVL6 ratios (18:0/16:0, 18:1n7/16:1n7) of fatty acids in phospholipids. All values were normalized to coconut diet representing 1, and statistical difference was accepted for *p* < 0.05, *COC (C)* coconut fat, *MAR (M)* margarine, *OLI (O)* olive oil, *SUN (S)* sunflower oil, *FIS (F)* fish oil

The main ELOVL6 product 18:1n7 was found in the highest concentrations in the fish oil diet and lowest in the coconut fat diet (Fig. 2a). The summarized ratio of ELOVL6 substrates vs. products was highest in the coconut fat diet and lowest in the fish oil and margarine diets (Fig. 2b). The specific ratio of 18:0/16:0 was highest in the coconut fat diet and lowest in the margarine diet (Fig. 2c). The 18:1n7/16:1n7 ratio was highest in the sunflower oil and lowest in the fish oil diets (Fig. 2c).

##### Effects of diets on the hepatic gene expression of ELOVL6

The hepatic gene expression of ELOVL6 was significantly lower after the fish oil diet compared with the margarine and olive oil diets. Furthermore, there was a significant difference between the high ELOVL6 expressions in the margarine diet compared with the olive oil supplementation diet (Fig. 2d).

##### Relative concentrations of fatty acids and fatty acid ratios in phospholipids

Concentrations of 18:0, 16:0, and 16:n7 in phospholipids were described previously for SCD1 (1.1.3.). The highest 18:1n7 fatty acid concentration in plasma phospholipids was found in animals fed the coconut fat diet; all other diets revealed significantly lower 18:1n7 levels (*p* < 0.05). The lowest level of 18:1n7 was found in the fish oil supplementation group, which was significant lower compared with coconut fat, margarine and olive oil supplementation group (*p* < 0.05, Fig. 2e). The ratio of substrates vs. products of ELOVL6 in plasma phospholipids was highest in the coconut fat diet (significantly higher vs. margarine and fish oil diet) and lowest in plasma phospholipids of the fish oil diet (significantly lower compared with the coconut and sunflower oil diet) (Fig. 2f). The ELOVL6-specific ratio of 18:0/16:0 was highest in plasma phospholipids of the sunflower oil supplementation group and lowest in the fish oil supplementation group (Fig. 2g). The 18:1n7/16:1n7 ratio was highest after the olive oil supplementation diet (significantly higher compared with the margarine and fish oil diet groups) and lowest in the fish oil supplementation group (significant when compared with the coconut fat, olive oil, and sunflower oil diet groups (Fig. 2g).

In summary, the single fatty acids 16:0, 18:0, 16:1n7, and 18:1n7 supplemented in the diet did not reflect the content in plasma phospholipids. The fatty acid ratios of plasma phospholipids reflected the hepatic gene expression of ELOVL6 only in the fish oil diet group (Fig. 2d, f).

### Hepatic gene expression of CYP26A1

**Fig. 3:**
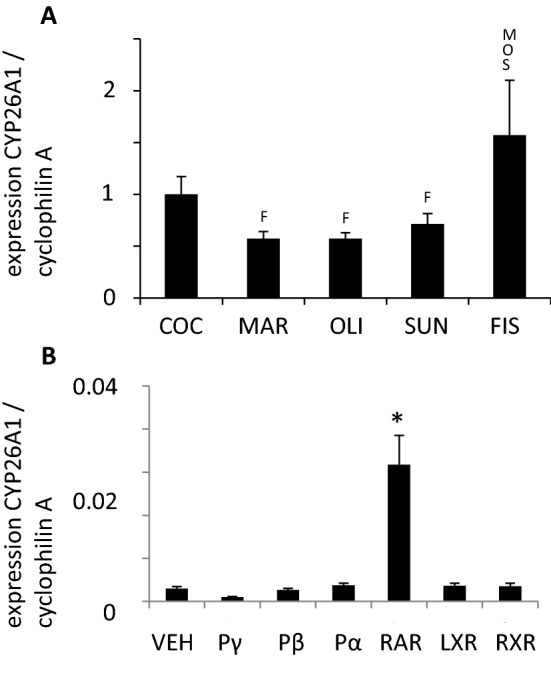
**a** Hepatic gene expression of CYP26A1. All values were normalized to coconut diet representing 1, statistic difference was accepted for *p* < 0.05, *COC (C)* coconut fat, *MAR (M)* margarine, *OLI (O)* olive oil, *SUN (S)* sunflower oil, *FIS (F)* fish oil. **b** Hepatic gene expression of CYP26A1 after administration of vehicle (VEH) and additional synthetic and specific ligands of the nuclear hormone receptor family [Pγ (PPARγ), Pβ (PPARβ/δ) or Pα (PPARα), RXR, and LXR]. Statistical difference was accepted for *p* < 0.05

The hepatic gene expression of CYP26A1 was significantly higher in the fish oil diet compared with the margarine, olive oil, and sunflower oil diets (Fig. 3a). In addition, CYP26A1 was significantly increased exclusively after administration of a specific RAR ligand and not by any other specific ligand of the nuclear hormone receptor family such as PPARα, β/δ or γ, RXR and LXR (Fig. 3b).

## Discussion

We investigated the impact of supplementing diets with different sources of fat and oils and the effect of this supplementation on the hepatic gene expression of major lipid-metabolizing enzymes which control plasma phospholipid composition. Our research question was to elucidate whether MUFA and SAFA contents in plasma phospholipids is affected by diet or by gene expression of metabolizing enzymes [8, 42]. For this purpose, we examined the hepatic gene expression of specific fatty acid-metabolizing enzymes SCD1 and ELOVL6 in the liver of mice and analyzed the fatty acid composition of diets and plasma phospholipids. To allow a comprehensive comparison between diets, we normalized all results for relative fatty acid content in the diet, hepatic gene expression, and phospholipid composition against the diet group supplemented with coconut fat. This allowed us to compare changes between the groups. We calculated product vs. substrate ratios specific for the lipid-metabolizing enzymes in both diet and phospholipids, and these were displayed together with the hepatic gene expression of the metabolic enzyme.

### SCD1 and ELOVL6 and their specific fatty acids in phospholipids

**Fig. 4:**
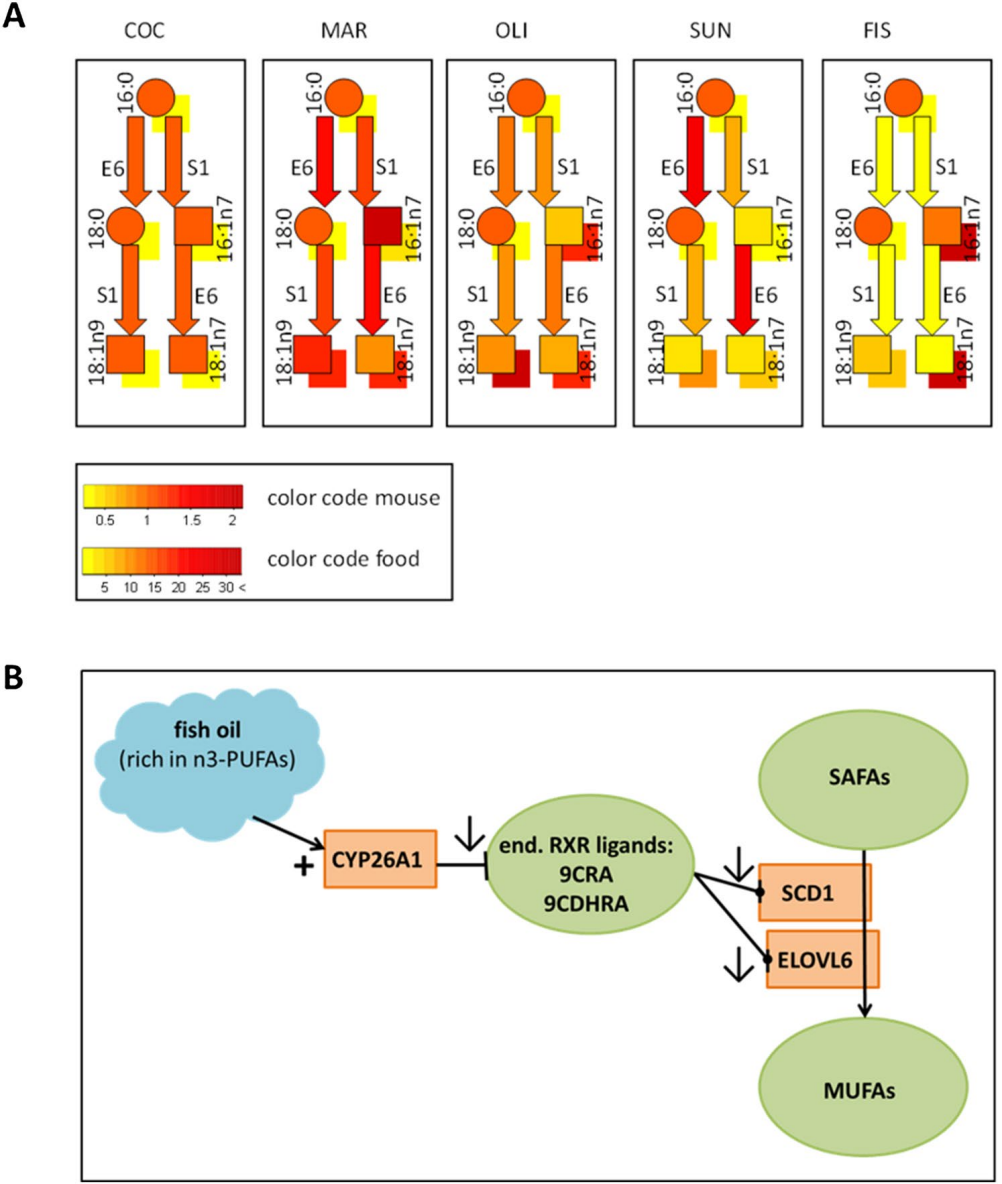
**a** Metabolic pathways of SCD1 (S1) and ELOVL6 (E6) mediation of 16:0 and 18:0–16:1n7, 18:1n7 and 18:1n9 in plasma phospholipids and dietary fats effects. The supplemented dietary fat is shown as a shadow behind the individual fatty acid in plasma phospholipids. **b** Proposed mechanisms for fish oil supplementation and modification of hepatic CYP26A1 expression and concomitant effects on SAFA to MUFA synthesis via SCD1 and ELOVL6 expression. *COC* coconut fat, *MAR* margarine, *OLI* olive oil, *SUN* sunflower oil, *FIS* fish oil

Our results clearly showed that the content of MUFAs and SAFAs in phospholipids is highly dependent on the hepatic gene expression of the lipid-metabolizing enzymes SCD1 and ELOVL6 and to a lesser extent on the ingestion of MUFA or SAFA (summarized in Fig. 4a). We found that the ratio 18:1n9/18:0 highly reflects the hepatic expression of SCD1, however this was only partly observed in the ratio 16:1n7/16:0. Our observation confirms previous findings in human plasma [43, 44]. In our study, 16:1n7/16:0 was further directly affected by fatty acids highly present in the fish oil diet. This fish oil diet contained high amounts of 16:1n7 and consumption leads to a high amount of 16:1n7 in plasma phospholipids in mice which could be an explanation for the elevated 16:1n7/16:0 ratio (Fig. 1a, e). In addition to this, we observed that in animals fed the fish oil diet, the hepatic expression of SCD1 was significantly lower compared with other dietary fats which explains the low levels of 18:1n9 incorporated into plasma phospholipids and the reduced 18:1n9/18:0 ratio. On the other hand, higher 18:1n9/18:0 and 16:1n7/16:0 ratios were observed in phospholipids of animals fed the coconut fat and margarine diets. These results highly reflect the increased hepatic gene expression of SCD1 following the coconut and margarine diets in comparison with those animals fed fish oil diet.

We also investigated specific fatty acid ratios in plasma 18:0/16:0 and 18:n7/16:1n7, because they have the potential to reflect the ELOVL6 hepatic gene expression [21]. Knock-out models of ELOVL6 have shown the importance of this enzyme for the elongation of fatty acids derived from diet or de novo fatty acid synthesis to provide sufficient amounts of 18:0 and 18:1n9 in the liver of mice [21]. Thereby, specific fatty acid ratios of 18:0/16:0 and 18:n7/16:1n7 were suggested by Matsuzaka et al. to reflect the ELOVL6 enzymatic reaction [21]. In one of our previous studies, we induced an overexpression of ELOVL6 in the liver by administration of specific ligands to activate the NHRs, LXR, and RXR, which induced the ELOVL6 hepatic gene expression leading further to an increase in the specific fatty acid ratios of ELOVL6 in liver and plasma [24]. In this study, we showed that feeding mice with fish oil, margarine, olive oil, and sunflower oil diets which contained high levels of 18:1n7 (Fig. 2a) resulted in low plasma phospholipid levels of this specific fatty acid (Fig. 2e). This result was more pronounced in the fish oil diet group, as the hepatic expression of ELOVL6 was significantly lower, which could further explain the low fatty acid ratio of 18:n7/16:1n7 observed in plasma phospholipids of this diet group. The low ratio of 18:0/16:0 in phospholipids of the fish oil diet group which was observed seems to result from the low hepatic gene expression of ELOVL6. Other suppressing effects by diet on ELOVL6 gene expression have been previously reported by our group. High fat diets rich in n6-PUFAs also suppressed ELOVL6 hepatic gene expression in a dose-dependent manner [22]. This suppression led to reduced enzymatic products of ELOVL6 in phospholipids [22].

The fish oil supplementation diet significantly reduced the hepatic gene expression of SCD1 and ELOVL6 in comparison with other sources of fat. This resulted in lower levels of MUFAs and higher levels of SAFAs in plasma phospholipids (summarized in Fig. 4a). The depletion and down regulation of SCD1 and ELOVL6 gene expression (indicating low levels of phospholipid MUFAs and high levels of SAFAs) might contribute to the protective mechanisms against diet-induced obesity, hepatic steatosis, and/or type 2 diabetes, as was shown in SCD1 and ELOVL6 knock-out models [45, 46]. This postulates different beneficial effects of fish oil/n3-PUFA diets besides functioning as precursors for anti-inflammatory and pro-resolving lipid mediators [21, 23, 47].

### Gene regulation connecting the fatty acid and retinoid pathway

Although the hepatic gene expression of SCD1 and ELOVL6 by dietary supplemented fat and oils has been well investigated, it is still not clear why a fish oil diet rich in n3-PUFAs (20:5n3, 22:6n3) and in n6-PUFAs results in a comparable low hepatic expression of these enzymes [31, 48–50]. For SCD1 and ELOVL6, we previously showed that the application of a synthetic LXR or RXR ligand can strongly induce hepatic SCD1 and ELOVL6 expression [24]. A retinoic acid is proposed as an endogenous RXR ligand [51], which our group found recently to be most likely 9-*cis*-13,14-dihydroretinoic acid (9CDHRA) [26, 52]. Afterwards, retinoic acid can be oxidized by the CYP26A1 and becomes inactive for receptor binding [29, 53]. Interestingly, 22:6n3 also has an inductive effect on the gene expression of CYP26A1 [30] via an unknown mechanism of action. We found that the fish oil-supplemented diet high in 22:6n3 strongly induced hepatic gene expression of CYP26A1 (Fig. 3a). The increased CYP26A1 expression might further catabolize 9-*cis* retinoic acid (9CRA) and 9CDHRA and reduce their availability as endogenous RXR ligand [26]. This might lead to reduced RXR-mediated signaling which may suppress hepatic SCD1 and ELOVL6 gene expression as observed in our experiment. The application of synthetic ligands to activate NHR showed that just the specific RAR ligand was able to strongly and significantly induce hepatic CYP26A1 expression (Fig. 3b). It seems likely that fish oil supplementation may mediate RAR-supported CYP26A1 expression via still unknown pathways (Fig. 4b). The activation of RAR-mediated signaling by a fish oil diet rich in n3-PUFAs may thereby be a major regulator of gene expression of the fat metabolizing target genes SCD1 and ELOVL6. Our findings suggest a potential new and important regulatory mechanism in which a fish oil diet rich in n3-PUFAs can alter lipid metabolism and might explain protective effects of PUFAs in obesity and diabetes [23] (summarized in Fig. 4b). We have used a mouse model with hepatic fatty acid-metabolizing enzymes that are also expressed in human liver [54–57]; for example, PPARs hepatic expression is similar in mice and humans [58]. PPARγ expression and modulation is crucial in adipogenesis, lipid metabolism, and inflammation. PPARγ expression is modulated by dietary components belonging to the lipid family which undergoes extensive metabolism in the liver and acts as an inductor or suppressor of its expression [59]. The interaction between dietary fat type, circulating fatty acid patterns, and hepatic enzyme expression is likely to result in altered liver metabolism. This might have other major metabolic implications. For example, fish oil supplementation compared to olive oil increased beta-oxidation rate resisting the inhibitory effect of insulin which did not occur in olive oil supplementation. Fish oil also reduced fatty acid incorporation into triglycerides and secretion of VLDL triglycerides [60]. However, the applicability of our findings for humans remains speculative. We used different regimes of fat supplementation in controlled animal feeding studies which might not be relevant to human diets; thus, further studies using human cell lines, ex vivo human tissues, or human dose relevant clinical supplementation trials are needed for translational purposes.

## Conclusion

We found that the MUFA/SAFA contents in mouse plasma phospholipids are mainly reflected and likely directly regulated by dietary stimuli altered hepatic gene expression of major lipid-metabolizing enzymes SCD1 and ELOVL6. In summary, MUFAs and SAFAs present in phospholipids are dependent on the transcriptional induction at hepatic gene expression level, and to lesser extent, these respond to dietary ingestion and further incorporation in the body.

## Abbreviations

SCD1: Stearoyl-Coenzyme A desaturase 1
ELOVL6: Elongase 6
MUFA: Monounsaturated fatty acid
SAFA: Saturated fatty acid
CYP26A1: Cytochrome P450 26A1
RXR: Retinoid X receptor
RAR: Retinoic acid receptor
